# Lipoprotein Metabolism Indicators Improve Cardiovascular Risk Prediction

**DOI:** 10.1371/journal.pone.0092840

**Published:** 2014-03-25

**Authors:** Daniël B. van Schalkwijk, Albert A. de Graaf, Evgeni Tsivtsivadze, Laurence D. Parnell, Bianca J. C. van der Werff-van der Vat, Ben van Ommen, Jan van der Greef, José M. Ordovás

**Affiliations:** 1 Department of Microbiology and Systems Biology, TNO, Zeist, The Netherlands; 2 Amsterdam University College, Amsterdam, The Netherlands; 3 Department of Risk Analysis for Products in Development, TNO, Zeist, The Netherlands; 4 The Nutrition and Genomics Laboratory, JM-USDA Human Nutrition Research Center on Aging at Tufts University, Boston, Massachusetts, United States of America; 5 Sino-Dutch Centre for Preventive and Personalized Medicine, Leiden Amsterdam Centre for Drug Research, Analytical Sciences Division, Leiden, The Netherlands; Medizinische Hochschule Hannover, Germany

## Abstract

**Background:**

Cardiovascular disease risk increases when lipoprotein metabolism is dysfunctional. We have developed a computational model able to derive indicators of lipoprotein production, lipolysis, and uptake processes from a single lipoprotein profile measurement. This is the first study to investigate whether lipoprotein metabolism indicators can improve cardiovascular risk prediction and therapy management.

**Methods and Results:**

We calculated lipoprotein metabolism indicators for 1981 subjects (145 cases, 1836 controls) from the Framingham Heart Study offspring cohort in which NMR lipoprotein profiles were measured. We applied a statistical learning algorithm using a support vector machine to select conventional risk factors and lipoprotein metabolism indicators that contributed to predicting risk for general cardiovascular disease. Risk prediction was quantified by the change in the Area-Under-the-ROC-Curve (ΔAUC) and by risk reclassification (Net Reclassification Improvement (NRI) and Integrated Discrimination Improvement (IDI)). Two VLDL lipoprotein metabolism indicators (VLDL_E_ and VLDL_H_) improved cardiovascular risk prediction. We added these indicators to a multivariate model with the best performing conventional risk markers. Our method significantly improved both CVD prediction and risk reclassification.

**Conclusions:**

Two calculated VLDL metabolism indicators significantly improved cardiovascular risk prediction. These indicators may help to reduce prescription of unnecessary cholesterol-lowering medication, reducing costs and possible side-effects. For clinical application, further validation is required.

## Introduction

The Framingham Risk score predicts cardiovascular risk based on six variables: age, diabetes, smoking status, treated and untreated systolic blood pressure, total cholesterol, and HDL (High Density Lipoprotein) cholesterol [1]. Newer lipoprotein measurement methods have attempted to improve risk prediction by quantifying lipoprotein subclasses by size [2]–[7] or density [8] range. However, the lipoprotein size information is little used in clinical practise so far, because its relation to cardiovascular risk is unclear. However, the lipoprotein size information contains implicit information about lipoprotein metabolism, which causes the size distribution. This metabolic information may be relevant for the prediction of cardiovascular disease.

We have developed a computational model to analyze measured lipoprotein subclass profiles in terms of the underlying metabolic activity [9]–[12]. Briefly, lipoproteins transport lipids, mainly triglycerides and cholesterol, through the bloodstream. The model includes Apolipoprotein B (ApoB)-containing lipoprotein particles ranging from large Very Low Density Lipoprotein (VLDL) through the smaller Intermediate Density Lipoprotein (IDL) and Low Density Lipoprotein (LDL) particles. Lipoprotein particles are produced by the liver, they lose fat to different tissues and become smaller in the lipolysis process, and they are finally taken up by the liver again. The main proteins responsible for the lipolysis process are Hepatic Lipase (HL) in the liver and Lipoprotein Lipase (LPL) in other tissues. The model can calculate ratios of lipoprotein production, lipolysis, and uptake processes from a single lipoprotein profile measurement; we call these ratios ‘lipoprotein metabolism indicators’.

ApoB-containing lipoproteins are proatherogenic because an accumulation of especially small dense LDL particles may lead to plaque formation in veins and arteries. Growing plaques may over time lead to CVD. Small dense LDL particles can form when the liver does not clear LDL particles from the bloodstream effectively. This is a metabolic disorder of the liver, that will also have an effect on VLDL, the metabolic precursor of LDL, because when overloaded the liver will also take up less VLDL, and perhaps produce more VLDL to lose excess fat. Therefore, we hypothesized that adding metabolic information in the form of lipoprotein metabolism indicators to conventional risk factors can improve cardiovascular risk prediction. We evaluated this hypothesis for subjects from the Framingham offspring cohort.

## Methods

### Study Sample and Risk Factors

In this study we used measured information from subjects studied in the 4^th^ examination of the Framingham Heart Study Offspring cohort, as recorded in the database of Genotypes and Phenotypes (dbGaP) [13]. Subjects were included when they had no history of cardiovascular disease, gave written informed consent for general research use, had complete NMR lipoprotein profiles recorded, and had a complete record of conventional cardiovascular risk factors. Cardiovascular events were carefully recorded during the follow-up period for all subjects.

### Computational Modeling

We applied the Particle Profiler computational model [9], [10] to NMR lipoprotein profiles [14]. Profiles were based on the original NMR measurements, to which Liposcience’s LP3 algorithm was applied. Slight modifications to the previously published Particle Profiler [12] fitting procedure can be found in Text S1 (Methods). We calculated ratios of all modeled processes (lipoprotein production, total lipoprotein lipolysis, HL lipolysis, LPL lipolysis, liver lipoprotein attachment, liver lipoprotein uptake) in each of three sets of lipoprotein size ranges (VLDL through LDL, VLDL only, IDL through LDL). The calculated ratios of modelled processes are lipoprotein metabolism indicators that serve as candidate diagnostics.

### Outcomes

Subjects who experienced a general cardiovascular event, as defined by the Framingham Heart Study [14], within 10 years after the NMR measurements, were designated as ‘cases’, all others as ‘controls’. The Framingham definition includes coronary death, myocardial infarction, coronary insufficiency, angina, ischemic stroke, hemorrhagic stroke, transient ischemic attack, peripheral artery disease, and heart failure.

### Statistical Analysis

We used a statistical learning algorithm (a nonlinear L2-norm support vector machine [15], [16]) to correlate predictor variables with the Cardiovascular Disease (CVD) outcome. This analysis was carried out in order to identify the most predictive ‘lipoprotein metabolic indicator' diagnostics, and evaluate their performance.

We grouped the predictor variables into three datasets: 1. conventional cardiovascular risk parameters, without cholesterol (*see* Table 1); 2. conventional cholesterol parameters (*see* Table 1) and 3. lipoprotein metabolism indicators (*see* Text S1 (Methods)). A detailed explanation of the procedure we used for constructing the multivariate model is provided in Text S1 (Methods). In summary, in order to obtain a model similar to the Framingham Risk Score, we selected the six most predictive variables from dataset 1, the two most predictive markers from dataset 2, and further markers from dataset 3. In the first phase, using dataset 1, we included ‘age’ and ‘gender’ in the model. We then added in succession those variables that contributed most to improving predictive performance of the model, measured as the area under the Receiver Operating Characteristic (ROC) curve (or C-statistic) [17]. The area under the ROC curve is the conventional statistic used for comparing the predictive performance of diagnostics. A procedure that successively adds the best predicting variables is frequently referred to as “forward variable selection” (see e.g. [18]). Having selected the biomarkers from dataset 1, we proceeded in a similar manner with datasets 2 and 3, consecutively adding the most predictive variables to the model. We added markers from dataset 3 that gave a substantial improvement in ROC prediction and that were not correlated with markers already in the model (r^2^<0.25); this procedure led to inclusion of two additional markers from dataset 3. For comparison, we also included a dataset with the selected markers from dataset 1, plus total and HDL cholesterol. We used a separate training and test-set for marker selection, but evaluated the final result using the complete dataset. All multivariate analyses were performed using Numerical Python.

**Table 1:** Markers present in raw datasets 1 and 2, the third dataset contains lipoprotein metabolic ratios, defined in Text S1 (Methods).

**Classical risk variables (dataset 1)**
Age
Sex
Systolic blood pressure physician 1
Diastolic blood pressure physician 1
Systolic blood pressure physician 2
Diastolic blood pressure physician 2
Systolic blood pressure nurse
Diastolic blood pressure nurse
Cigarettes per day
Inhales
Smokes sigars
Smokes pipe
Spouse smokes
BMI
Blood pressure medication
Glucose
**Cholesterol markers (dataset 2)**
Total cholesterol
HDL cholesterol
VLDL cholesterol (NMR)
LDL cholesterol (NMR)
HDL cholesterol (NMR)

The results of the multivariate analyses are various predictive models including different diagnostic markers. The CVD risk predictions of these models then need to be compared using suitable statistics. We compared the area under the ROC curve of the various models (ΔAUC) using the method by de Long and a binomial exact test, calculated in MedCalc, version 11.5.1.0. Also, we used Platt’s algorithm to transform the predictions computed by SVM into class probabilities for computing reclassification statistics [19], [20]. Reclassification was quantified using the ‘Net Reclassification Improvement’ (NRI) using 6% and 20% risk cutoffs for the ‘medium’ and ‘high’ risk classes and the ‘Integrated Discrimination Improvement’ (IDI, a risk cutoff-independent method) as suggested by Pencina [21]. The first ROC analysis is the classical comparison of diagnostic power. The reclassification comparison is more sensitive and gives more clinically relevant information, because it measures how people are redistributed over risk categories using the new diagnostics, and evaluates whether that change was correct.

## Results

### Baseline Characteristics

Of the 2142 selected subjects 145 cases and 1836 controls were found to have a complete record of all relevant parameters and thus were included in the analysis. Baseline characteristics of the subjects are shown in Table 2. The mean age was 49±9 years, 52% was female.

**Table 2:** Baseline characteristics of the subjects.*

Characteristic	Men (N = 946)	Women (N = 1035)
Mean age – yr	49.2±9.3	49.5±9.0
Cholesterol – mg/dl		
Total	204±36	205±40
HDL	44±11	56±15
Blood pressure – mm Hg		
Systolic (nurse)	127±16	122±19
Diastolic (nurse)	80±10	75±10
Blood pressure medication – no. (%)	126 (13.3)	128 (12.4)
Body-mass index	27.6±3.8	25.8±5.1
Smoking		
Smokes and inhales	201 (21.2)	219 (21.2)
Cigarettes per day	5.2±12.1	4.2±9.6
Smokes cigars – no. (%)	46 (4.9)	2 (0.2)
Smokes pipe – no. (%)	28 (3.0)	0 (0)
Spouse smokes – no. (%)	344 (36.4)	450 (43.5)
Glucose – mg/dl	95±18	91±22

* Plus-minus values are means ± SD. To convert the values for cholesterol to millimoles per liter, multiply by 0.02568. The body-mass index is the weight in kilograms divided by the square of the height in meters. HDL denotes high-density lipoprotein.

### Multivariate Models

The variables included in the final multivariate models are shown in Table 3. The first selected lipoprotein metabolism indicators was 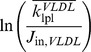, which we call the ‘VLDL Extrahepatic lipolysis indicator’ or VLDL_E_. This indicator is a ratio between the VLDL lipolysis rate related to lipoprotein lipase (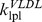 ) and the influx of particles due to production in the liver and lipolysis of larger particles (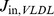). The second selected lipoprotein metabolism indicator 
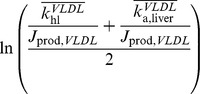
, which we call the ‘VLDL Hepatic turnover indicator’ or VLDL_H_, is the average of two ratios: that between the rate constant of hepatic VLDL lipolysis (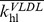) and the VLDL particle production flux (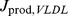), and that between the rate of VLDL attachment to the liver (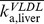) and the VLDL particle production flux. Further explanation of the mathematical notation of these indicators can be found in Text S1 (Methods).

**Table 3:** Variables included in final multivariate models.

Conventional markers without cholesterol	Conventional markers	LDLc	LDLc + HDLc	LDLc + HDLc + VLDL_E_ + VLDL_H_
Age	Age	Age	Age	Age
Sex	Sex	Sex	Sex	Sex
Cigarettes per day	Cigarettes per day	Cigarettes per day	Cigarettes per day	Cigarettes per day
Blood pressure medication	Blood pressure medication	Blood pressure medication	Blood pressure medication	Blood pressure medication
Systolic blood pressure (nurse)	Systolic blood pressure (nurse)	Systolic blood pressure (nurse)	Systolic blood pressure (nurse)	Systolic blood pressure (nurse)
Glucose	Glucose	Glucose	Glucose	Glucose
	Total Cholesterol	LDL cholesterol	LDL cholesterol	LDL cholesterol
	HDL cholesterol		HDL cholesterol	HDL cholesterol
				VLDL Extrahepatic lipolysis indicator
				VLDL Hepatic turnover indicator

### ROC analysis of Multivariate Models

Tables 4 and 5 show the results of a Receiver-Operating-Characteristic (ROC) analysis for general cardiovascular disease. Table 4 displays the area under the curve, its improvement over a predictor drawn at random, and a percentage incremental improvement of the last statistic. Results of the statistical analyses comparing the curves are shown in Table 5. Our method significantly improved CVD prediction over accepted risk markers, as measured by the Area-Under-the-ROC-Curve (ΔAUC). The improvement of our model versus a model with classical Framingham risk markers, including total cholesterol and HDLc, was ΔAUC = 0.0177 with p = 0.0055. The improvement of our model versus a model including LDLc and HDLc was ΔAUC = 0.0150 with p = 0.0067. In comparison, the model including LDLc and HDLc did not significantly improve risk prediction over the model including total cholesterol and HDLc, with ΔAUC = 0.00268, and p = 0.6003. As expected, adding total and HDL cholesterol to other classical Framingham risk factors did significantly improve risk prediction, with ΔAUC = 0.0354 and p = 0.0003. The statistical test thus showed that adding lipoprotein metabolism indicators to a model that includes existing cardiovascular risk factors significantly improved the area under the ROC curve for this population, with respect to conventional risk markers.

**Table 4:** Areas under the ROC curve for the cross-validated multivariate models and their improvement versus a random predictor.

Model	AUC	SE	AUC improvement from random	% incremental AUC improvement from random
Conventional, no cholesterol	0.759	0.0204	0.259	0.0
Conventional	0.795	0.0193	0.295	12.2
LDLc	0.791	0.0192	0.291	11.0
LDLc + HDLc	0.797	0.0192	0.297	12.8
LDLc + HDLc + VLDL_E_ + VLDL_H_	0.812	0.0192	0.312	17.0

**Table 5:** Statistical analysis of areas under the ROC curve for the cross-validated multivariate models.

Model 1	Model 2	Difference AUC ROC curve	Standard Error	P value
Conventional no cholesterol	Conventional	0.0354	0.00979	0.0003
Conventional no cholesterol	LDLc	0.0323	0.00991	0.0011
Conventional	LDLc + HDLc	0.00268	0.00512	0.6003
Conventional	LDLc + HDLc + VLDL_E_ + VLDL_H_	0.0177	0.00637	0.0055
LDLc	LDLc + HDLc	0.00580	0.00566	0.3055
LDLc	LDLc + HDLc + VLDL_E_ + VLDL_H_	0.0208	0.00735	0.0047
LDLc + HDLc	LDLc + HDLc + VLDL_E_ + VLDL_H_	0.0150	0.00552	0.0067

### Reclassification analysis

Table 6 shows the results of the reclassification analysis. Risk reclassification, using low, middle, and high risk classes, and also using the category independent methods was significantly improved when including LDLc, HDLc, and VLDL metabolism indicators. The improvement of the model including VLDL metabolism indicators versus the model including classical Framingham risk markers was quantified as NRI = 0.090, with p = 0.014; for the category independent method IDI = 0.051, with p<0.0001. The improvement of the model including VLDL metabolism indicators versus the model including LDLc and HDLc was quantified as NRI = 0.0828, with p = 0.013; for the category independent method IDI = 0.040, p = 0.0004. In comparison, the model including total cholesterol and HDLc versus that including LDLc and HDLc was nonsignificant, with NRI = 0.008 and IDI = 0.011. Adding total and HDL cholesterol to non-cholesterol Framingham risk factors did give a significant improvement, with NRI = 0.111 and p = 0.009; for the category independent method IDI = 0.040, p<0.0001. Lipoprotein metabolism indicators therefore add reclassification power to the NMR lipoprotein profile.

**Table 6:** Reclassification analysis: Net Reclassification Improvement (NRI) and integrated discrimination improvement (IDI) when comparing the cross-validated multivariate models using all subjects in the dataset.

Model 1	Model 2	NRI	Standard Error	P value	% of Events correctly reclas-sified (n = 145)	Event P-value	% of Non-events correctly reclas-sified (n = 1836)	Nonevent P-value
Conventional no cholesterol	Conventional	0.1111	0.0418	0.0088	8%	0.0455	3%	0.002
Conventional no cholesterol	LDLc	0.0830	0.0386	0.0327	6%	0.1441	3%	0.0021
Conventional	LDLc + HDLc	0.0080	0.0339	0.8135	1%	0.8348	0%	0.8802
Conventional	LDLc + HDLc + VLDL_E_ + VLDL_H_	0.0902	0.0366	0.0143	5%	0.1779	4%	<.0001
LDLc	LDLc + HDLc	0.0356	0.0307	0.2489	3%	0.2513	0%	0.8751
LDLc	LDLc + HDLc + VLDL_E_ + VLDL_H_	0.1178	0.0376	0.0020	8%	0.0411	4%	<.0001
LDLc + HDLc	LDLc + HDLc + VLDL_E_ + VLDL_H_	0.0828	0.0330	0.0127	4%	0.2008	4%	<.0001
Model 1	Model 2	Abso-lute IDI	Standard Error	P value	Proba-bility change for events (n = 145)	Proba-bility change for non-events (n = 1836)	Relative IDI	
Conventional no cholesterol	Conventional	0.0402	0.0096	<.0001	0.0372	–0.0029	0.3784	
Conventional no cholesterol	LDLc	0.0362	0.0102	0.0004	0.0335	–0.0026	0.3410	
Conventional	LDLc + HDLc	0.0107	0.0077	0.1652	0.0099	–0.0008	0.0733	
Conventional	LDLc + HDLc + VLDL_E_ + VLDL_H_	0.0506	0.0120	<.0001	0.0469	–0.0037	0.3461	
LDLc	LDLc + HDLc	0.0147	0.0045	0.0010	0.0136	–0.0011	0.1033	
LDLc	LDLc + HDLc + VLDL_E_ + VLDL_H_	0.0546	0.0124	<.0001	0.0506	–0.0040	0.3836	
LDLc + HDLc	LDLc + HDLc + VLDL_E_ + VLDL_H_	0.0399	0.0113	0.0004	0.0370	–0.0029	0.2541	

In addition, we calculated NRI reclassification statistics for subjects classified as at ‘Intermediate risk’ when using Framingham risk markers (Table 7). These subjects would be eligible for drug treatment in primary prevention. The analysis shows how many subjects not experiencing events would not need treatment, and how many experiencing events would be put on more intensive treatment when using the new diagnostics. The NRI was 0.15 (p = 0.0481) when comparing these conventional markers to LDLc and HDLc, and 0.37 (p<0.0001) when comparing them to the model including lipoprotein metabolism indicators. When looking at reclassified events separately (n = 48), the two mentioned methods improved classification by 6% and 13% respectively, but both improvements were non-significant. Importantly, there was a 9% reclassification improvement of non-events (n = 422) when including LDLc and HDLc, and a 25% reclassification improvement of non-events using lipoprotein metabolism indicators, both with p<0.0001. The study therefore shows that 25% of subjects that conventional Framingham risk factors would needlessly include in the ‘intermediate risk’ category, were reclassified to ‘low risk’ using lipoprotein metabolism indicators.

**Table 7:** Reclassification analysis of intermediate-risk subjects.

Model 1	Model 2	NRI	Standard Error	P value	% of Events correctly reclas-sified (n = 48)	Event P-value	% of Non-events correctly reclas-sified (n = 422)	Nonevent P-value
Conventional	LDLc + HDLc	0.15	0.08	0.0481	6%	0.4054	9%	<.0001
Conventional	LDLc + HDLc + VLDL_E_ + VLDL_H_	0.37	0.09	<.0001	13%	0.1336	25%	<.0001

## Discussion

This is the first study in which ‘lipoprotein metabolism indicators’ have been used for cardiovascular disease risk prediction. These diagnostics are ratios of lipoprotein production, lipolysis, and uptake processes derived from a single lipoprotein profile measurement using computational modelling. We demonstrate that incorporation of two lipoprotein metabolism indicators significantly improves CVD risk prediction as measured by the area-under-the-ROC-curve. Reclassification is also significantly improved over conventional risk markers. The most important predictor, the ‘VLDL Extrahepatic lipolysis indicator’ or VLDL_E_,is a ratio between the VLDL lipolysis rate related to lipoprotein lipase (LPL) and the influx of particles due to production in the liver and lipolysis of larger particles. As LPL mainly acts extrahepatically, this ratio gives information about the capacity of extrahepatic tissue to absorb triglycerides from VLDL particles in the fasting state. The second indicator, we call the ‘VLDL Hepatic turnover indicator’ or VLDL_H_, is the average of two ratios: that between hepatic VLDL lipolysis and VLDL production, and that between VLDL attachment to the liver and VLDL production. This combined ratio relates to the capacity of the liver to process VLDL particles, both through lipolysis and particle attachment to the liver. Inspection of the risk model (*see* Text S1, Results) shows that LDLc remains the most important lipoprotein-related predictor of CVD events. HDLc is an important risk modifier, especially when no blood pressure medication is used. When using blood pressure medication, VLDL_E_ becomes important; the lower this indicator, the slower incoming VLDL particles are lipolysed extrahepatically, the higher the risk. VLDL_H_ is most important for determining the border between low and medium risk, especially for men and when not using blood pressure medication; the lower VLDL_H_, the less hepatic VLDL turnover per produced particle, the higher the risk. These interpretations show that the new risk prediction can be understood in relation to lipoprotein pathophysiology and genetic variation (in *LPL* and other genes pertinent to VLDL processes).

Examining the reclassification of subjects that were classified as at ‘intermediate risk’ by Framingham risk factors is of special clinical significance. The intermediate risk group consists of those individuals that should be treated according to international guidelines [21]. Subjects that are reclassified move to either the high risk (more intensive treatment) or low risk (no treatment) groups. Our results show that a net 25% of subjects in this group that will not get cardiovascular disease after 10 years are moved to the low risk group. The reclassification of people with events to the high risk group was not significant, probably due to the low number of cases in this group (n = 48). In other words, there is a group of people that are classified as at 'intermediate risk' using the Framingham risk factors, but of whom we know with hindsight that they do not suffer from a cardiovascular event. When performing a diagnosis using VLDL_E_ and VLDL_H_, 25% of this subject group is reclassified to the low risk category, and these subjects would therefore not have to take the medication the guidelines prescribe for the intermediate risk category needlessly.

Extrapolating these results to clinical practice directly is not straightforward, most importantly because treatment decisions are most often made based on one or two parameters (such as LDLc and HDLc) and not based on a complete set of risk markers. However, because our multivariate model for the classical Framingham markers is already an improvement over the two-variable approach used in practice, a 25% improvement using our final risk model will most likely be an underestimate for a comparison with a two-variable approach used in the same population. Future studies will need to point out whether the 25% improvement can be validated in other populations, and whether a population with more CVD cases will also yield significant reclassification improvement for cases in the Intermediate risk category. Our methodology can be readily applied to any past studies in which NMR lipoprotein profiles have been measured. Possible subjects of further investigation includes determining risk in younger or older persons, differences in ethnic groups, and the benefits for secondary prevention. The Particle Profiler model can also derive lipoprotein metabolism indicators from other methods for measuring lipoprotein profiles [2]–[7]. Other future investigation can compare the results of modelling the data from these methods.

The current study has one technical limitation that deserves mention: the NMR spectra were recorded with an older version of the technology that is currently available. This limitation does not affect the method to derive lipoprotein metabolism indicators. Because of newer NMR methodology, the accuracy of lipoprotein metabolism indicators will increase in future studies.

In summary, in a sample of 1981 subjects from the Framingham offspring cohort, we found 2 lipoprotein metabolism indicators that together significantly improved general cardiovascular risk prediction, as quantified by the area under the ROC curve and by reclassification statistics. These indicators may help to reduce the number of people that unnecessarily take cholesterol-lowering medication, reducing costs and possible side-effects. Clinical application will require further validation of these findings.

## Supporting Information

Text S1 **Additional information on methods and results.**
(DOC)
