# Supplementary material for: Lipoprotein Metabolism Indicators Improve Cardiovascular Risk Prediction
Source: PLoS One. 2014 Mar 25;9(3):e92840. doi: 10.1371/journal.pone.0092840 (PMC3965475; doi:10.1371/journal.pone.0092840)
Supplement: Text S1 — Additional information on methods and results. (DOC) [file pone.0092840.s001.doc]

**Supporting Information S1**

Additional information on methods and results.

[Methods 2](#__RefHeading___Toc336960196)

[Modifications to the NMR data fitting procedure 2](#__RefHeading___Toc336960197)

[Complete dataset variables, prior to selection 2](#__RefHeading___Toc336960198)

[Classical and NMR variables 2](#__RefHeading___Toc336960199)

[Lipoprotein metabolism indicators 3](#__RefHeading___Toc336960200)

[Procedure used for constructing the multivariate model 3](#__RefHeading___Toc336960201)

[Results 5](#__RefHeading___Toc336960202)

[Inspection of the multivariate risk model 5](#__RefHeading___Toc336960203)

[References 6](#__RefHeading___Toc336960204)

# Methods

## Modifications to the NMR data fitting procedure

In a previous paper, we fitted the Particle Profiler model to NMR data measured in the GOLDN study[1]. In the GOLDN study, the NMR data contained three VLDL fractions. In the current study, the NMR data contained six VLDL fractions. Accordingly, we assigned new weights for fitting the data to VLDL fractions VLDL1 through VLDL4. Because of the small number of particles in VLDL fractions 5 and 6, we combined them and fitted them with the original weight for the ‘large VLDL’ fraction. Supporting Table 1 gives the weights for the new VLDL fractions, the other weights and the procedure are as previously described [1].

**Supporting Table S1: additional weights used for fitting the Particle Profiler model to NMR data**

| **Fraction name** | **Weight** |
| --- | --- |
| VLDL 1 | 0.1342 |
| VLDL 2 | 0.1276 |
| VLDL 3 | 0.1272 |
| VLDL 4 | 0.1310 |

## Complete dataset variables, prior to selection

### Lipoprotein metabolism indicators

All lipoprotein metabolism indicators can be expressed by the following general expressions:

$\ln\left(\frac{\overline{k_{rateprocess}^{range}}}{J_{\text{fluxprocess},range}}\right)$ expr. 1

$\ln\left(\frac{J_{\text{fluxprocess},range}}{\overline{k_{rateprocess}^{range}}}\right)$ expr. 2

$\ln\left(\frac{\overline{k_{rateprocess1}^{range}}}{\overline{k_{rateprocess2}^{range}}}\right)$ expr. 3

Where $\overline{k_{rateprocess}^{range}}$ indicates the particle size dependent rate of the process denoted by *rateprocess*, averaged per particle over the size range denoted by *range*. Where *rateprocess* is one of the following processes: *lpl* (LPL-related lipolysis), *hl* (HL-related lipolysis), *l* (total lipolysis, sum of LPL- and HL-related lipolysis), *u,liver* (liver uptake), or *a,liver* (liver attachment); and where *range* is one of the following size ranges: ILDL (IDL and LDL size range, 5-30 nm in the model), VLDL (VLDL size range, 30-80 nm in the model) or TOT (complete modeled size range, 5-80 nm in the model). Please note that the model does not include HDL, so these particles are not included in the smaller size range.

$J_{\text{fluxprocess},range}$ indicates the particle flux denoted by *fluxprocess* into the size range denoted by *range*. Where *fluxprocess* is one of the following processes: *prod* (direct production from the liver) or *in* (total influx due to both production and lipolysis of larger particles); and where *range* is one of the size ranges mentioned above.

The input dataset for constructing the multivariate prediction model consisted of all ratios indicated by expressions 1, 2, and 3. Because of the large number of zero entries, indicators with $\overline{k_{\text{lpl}}^{range}}$ or $J_{\text{prod},ILDL}$ in the denominator were excluded.

In addition, several averages of indicators were constructed, of the form:

$\ln\left(\frac{\frac{\overline{k_{rateprocessA}^{range}}}{J_{\text{fluxprocess},range}}+\frac{\overline{k_{rateprocessB}^{range}}}{J_{\text{fluxprocess},range}}}{2}\right)$ expr. 4

Where rateprocessA is one of the following processes: *lpl, hl,* or *l*; rateprocessB is either *a,liver* or *u,liver*. All other symbols are defined as described above. Indicators including $J_{\text{prod},ILDL}$ were excluded, because of the large number of zero entries.

These expressions result in 124 lipoprotein metabolism indicators from expressions 1, 2, and 3 and 30 from expression 4, which total 154 lipoprotein metabolism indicators.

## Procedure used for constructing the multivariate model

To construct a multivariate predictive model we used a state-of-the-art statistical machine learning algorithm, called ‘support vector machine’ (SVM) [2,3]. The method belongs to the class of so-called regularized kernel-based approaches that have been shown to outperform many standard classification methods. To conduct the experiment we scaled each variable to the range of [0,1] (note that lipoprotein metabolism indicators were then already log-transformed), we shuffled the dataset randomly, and we divided the data into two independent sets, that is, a training set (70%) and a validation set (30%). When training the algorithm there are number of hyper-parameters that have to be estimated to ensure good generalization performance. In our experiments we used a SVM algorithm with a squared loss function and a Gaussian kernel. Furthermore, we estimated the regularization parameter to prevent over-fitting on the training data. Optimal widths for the kernel function and regularization parameter were found via a cross-validation procedure on the training set. Once the parameters that led to the best predictive performance of the model were obtained, we retrained the algorithm on the complete training set and tested the performance on the separately reserved validation set. We evaluated predictive performance of the the model using area under ROC curve statistics (AUC) (C-statistic) [4].

We applied the above multivariate modeling approach in order to identify the best biomarkers. We used the validation set to estimate the biomarkers’ predictive performance. For this purpose we used a “forward-selection” procedure [5]. In order to obtain a model similar to the Framingham Risk Score, we selected the markers from three consecutive groups. The first group consists of ‘classical’ markers mentioned in Table 1 of the main article; from this set we selected two markers (age and sex) and let the algorithm identify four more markers. The second group consists of cholesterol markers mentioned in Table 1 of the main article; from this set we let the algorithm identify two markers. The third group consists of the log-transformed lipoprotein metabolism indicators; we let the algorithm select several markers, and decided how many to include based on AUC performance improvement and on lack of correlation with the already included markers (r^2^<0.25). After the addition of every marker we evaluated the area under the ROC curve on the validation set to evaluate how good the model performs with the set of markers selected so far.

This selection procedure led to the set of markers mentioned in Table 3 of the main article.

In order to test whether we could have performed biomarker identification with a simpler statistical method, we also used logistic regression with the same experimental set-up as described above. Supporting Table 2 shows the markers that were selected from group 1 using logistic regression. These results show that this second method selects four highly correlated blood pressure variables, very unlike the known Framingham Risk Score variables. Our SVM-based method does select variables corresponding to the Framingham Risk Score, indicating that we can trust the SVM method to produce more reliable results in this experimental set-up than logistic regression.

**Supporting Table S2: Markers selected from group 1 using logistic regression**

| Age |
| --- |
| Sex |
| Systolic blood pressure physician 1 |
| Diastolic blood pressure physician 1 |
| Systolic blood pressure physician 2 |
| Diastolic blood pressure physician 2 |

# Results

## Inspection of the multivariate risk model

In the body text we note that inspection of the risk model shows four points. Below we mention these points and refer to the illustrating figure.

1. LDLc remains the most important lipoprotein-related predictor of CVD events.
   1. Supporting Figure 1a shows how risk depends on LDLc, for a 60-year old male and female subject who do not use blood pressure medication, and have overall average values for all other predictor variables.
   2. The overall average values are: Cigarettes per day: 4.7; Systolic blood pressure: 124.2 (mm Hg); Glucose 5.0 (mg/dL); LDLc: 165.9 (mg/dL); HDLc: 60.0 (mg/dL); VLDL_E_: -4.43 (expressed as ln(fl/particle)) ; VLDL_H_: -3.40 (expressed as ln(fl/particle)).
   3. This figure show that a higher LDLc value leads to a higher risk in our model of the specified subjects.
2. HDLc is an important risk modifier, especially when no blood pressure medication is used.
   1. Supporting Figure 1b shows how risk depends on HDLc, for a 60-year old male and female subject who do not use blood pressure medication, using an LDLc on the low-to-medium risk boundary (LDLc: 130 mg/dL) and on the medium-to-high risk boundary (LDLc: 190 mg/dL). All other predictor variables have the population averages mentioned above.
   2. This figure shows that a lower HDLc leads to a higher risk in our model of the specified subjects. Lower LDLc lowers the overall risk.
3. When using blood pressure medication the VLDL Extrahepatic lipolysis indicator (VLDL_E_) becomes important; the lower the VLDL_E_, the less relative LPL turnover, the higher the risk.
   1. Supporting Figure 1c shows how risk depends on VLDL_E_, for a 60-year old male and female subject, who do use blood pressure medication. All other predictor variables have the population averages mentioned above.
   2. The figure shows that in subjects on blood pressure medication, a low VLDL_E_ dramatically increases the CVD risk, especially in men.
4. The VLDL Hepatic turnover indicator (VLDL_H_) is important for determining the border between low and medium risk, especially for men and when not using blood pressure medication; the lower the VLDL_H_ , the less relative hepatic turnover, the higher the risk.
   1. Supporting Figure 1d shows how risk depends on VLDL_H_ for a 60-year old male and female subject, who do not use blood pressure medication, and have LDLc on the low-to-medium risk boundary. All other predictor variables have the population averages mentioned above.
   2. The figure shows that a low VLDL_H_ increases the CVD risk in these subjects.

**Supporting Figure S1: Inspection of the multivariate risk model.**

Graphs are drawn up for a 60-year old male and female subject, who have overall population average risk factors for the other risk factors, except those specified in continuation. For A) CVD risk change with LDLc (mg/dL), subjects do not use blood pressure medication B) CVD risk change with HDLc (mg/dL), with LDLc at the low-medium risk border, and the medium-high risk border, subjects do not use blood pressure medication. C) CVD risk change with the VLDL Extrahepatic lipolysis indicator (expressed as ln(fl/particle)), subjects **do** use blood pressure medication. D) CVD risk change with the VLDL Hepatic turnover indicator (expressed as ln(fl/particle)), subjects do not use blood pressure medication, and have LDLc at the low-medium risk border.

# References

1. van Bochove C, van Schalkwijk DB, Parnell LD, Lai CQ, Ordovas JM et al. (2012) Clustering by plasma lipoprotein profile reveals two distinct subgroups with positive lipid response to fenofibrate therapy. PLoS One 7: e38072.

2. Hastie, Trevor, Tibshirani, Robert, and Friedman, J. H (2009) The elements of statistical learning: data mining, inference, and prediction. New York: Springer-Verlag.

3. Tsivtsivadze E, Pahikkala T, Boberg J, Salakoski T, Heskes T (2010) Co-Regularized Least-Squares for Label Ranking. In: Preference Learning. pp. 107-123.

4. Obuchowski NA (2005) ROC analysis. Am J Roentgenol 184: 364-372.

5. Guyon, Isabelle, Gunn, Steve, Nikravesh, Masoud, and Zadeh, Lotfi A. (2006) Feature Extraction: Foundations and Applications (Studies in Fuzziness and Soft Computing). New York: Springer-Verlag.
